# Nonphthalate Plasticizers in House Dust from Multiple Countries: An Increasing Threat to Humans

**DOI:** 10.1021/acs.est.2c08110

**Published:** 2023-02-22

**Authors:** Hongli Tan, Liu Yang, Xiaolin Liang, Diedie Huang, Xinhang Qiao, Qingyuan Dai, Da Chen, Zongwei Cai

**Affiliations:** † State Key Laboratory of Environmental and Biological Analysis, Hong Kong Baptist University, Hong Kong, SAR 999077, China; ‡ School of Environment, Guangdong Key Laboratory of Environmental Pollution and Health, Jinan University, Guangzhou 510632, China

**Keywords:** nonphthalate plasticizers, phthalate esters, house dust, multiple regions, human exposure

## Abstract

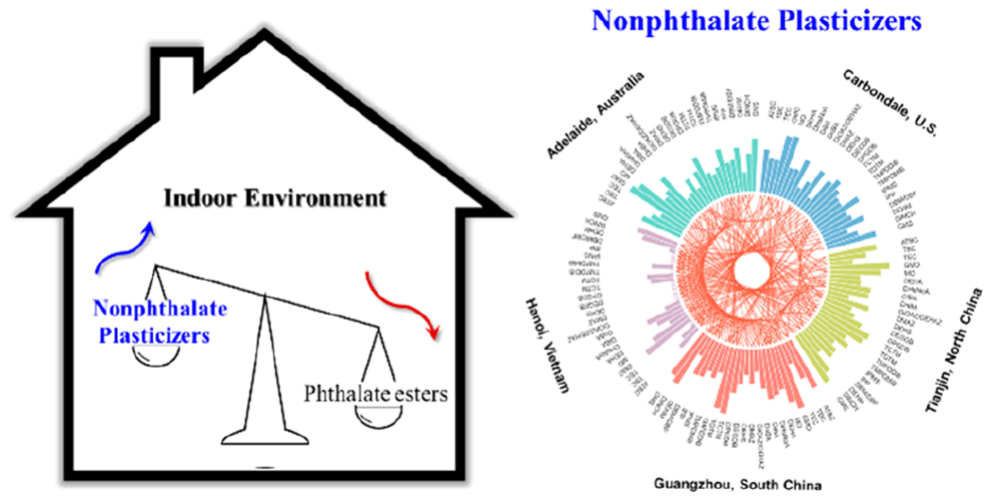

Along with the restrictions of phthalate esters (PAEs), a variety of nonphthalate plasticizers (NPPs) have been increasingly used for industrial needs. Knowledge remains limited on the environmental occurrences, fate, and human exposure risks of many emerging NPPs. In this study, we investigated a suite of 45 NPPs along with the major PAEs in house dust from five regions in the Asia-Pacific region and the United States. The findings clearly demonstrated ubiquitous occurrences of many NPPs in the home environment, particularly acetyl tributyl citrate (ATBC), tricapryl trimellitate (TCTM), trioctyl trimellitate (TOTM), glycerol monooleate (GMO), methyl oleate (MO), and diisobutyl adipate (DiBA). The median total concentrations of NPPs ranged from 17.8 to 252 μg/g in the study regions, while the mean ratios of ΣNPPs to ΣPAEs ranged from 0.19 (Hanoi) to 0.72 (Adelaide). Spatial differences were observed not only for the chemical abundances but also for the composition profiles and the hazard quotient (HQ) prioritization of individual chemicals. Although the current exposure may unlikely cause significant health risks according to the HQ estimation, potential exposure risks cannot be overlooked, due to the lack of appropriate toxic threshold data, the existence of additional exposure pathways, and possible cocktail effects from coexisting NPPs and PAEs.

## Abstract 2

A broad range of nonphthalate plasticizers was accurately identified and quantified in house dust from multiple regions, raising concerns about their global environmental distributions and human exposure risks.

## Introduction

Plasticizers represent a large group of chemical additives in order to increase the flexibility, transparency, durability, and longevity of polymers materials.^1,2^ As the most commonly used plasticizers, phthalate esters (PAEs) constitute about 80–85% of the global plasticizer market for use in polyvinyl chloride plastics.^3^ Until recently, the global production of PAEs even exceeded 8 million tons.^4^ However, large-scale applications over the past decades have resulted in ubiquitous occurrence of PAEs in global environment, wildlife, and humans.^4−6^ Numerous studies have demonstrated various toxic effects of PAEs, such as reproductive toxicity, carcinogenesis, cardiotoxicity, hepatotoxicity, and nephrotoxicity.^7,8^ Global environmental and health concerns have promoted many governments to formulate and enact strict regulations and bans on the use of PAEs, consequently resulting in increasing use of non-PAE plasticizers (NPPs).^3^

In 2017, the phthalate-free plasticizers accounted for 35% of global plasticizer consumption, up from 12% in 2005, and were expected to increase to 40% in 2022.^9,10^ NPPs are complex in chemical structures, mainly containing functional groups such as benzoate, sebacate, azelate, adipate, terephthalate, trimellitate, citrate, oleate, and a few others.^11^ Commonly used NPPs include di(2-ethylhexyl)terephthalate (DEHT), trioctyl trimellitate (TOTM), and diisononyl hexahydrophthalate (DINCH), di(2-ethylhexyl) adipate (DEHA), acetyl tributyl citrate (ATBC), and 2,2,4-trimethyl 1,3-pentanediol diisobutyrate (TMPDDiB).

Investigations on the NPPs’ environmental occurrences, fate, and human exposure risks remain overall limited compared to that for PAEs. Questions also remain largely unexplored as whether the NPPs are safe replacements or just “regrettable substitutions”.^12,13^ For example, DINCH, a nonphthalate plasticizer, was introduced into the market as a safe alternative to PAEs (e.g., di(2-ethylhexyl) phthalate, DEHPh) due to favorable toxicological profiles.^13^ However, recent studies implied otherwise. DINCH may disrupt metabolic and endocrine systems, cause oxidative stress, and damage DNA functions.^13−18^ Several in vitro and in vivo studies have indicated DINCH and DEHA exhibited toxic potencies (e.g., cytotoxicity, disruption of lipid metabolism in the mammary gland, and disruption of thyroid hormone activity) comparable to or even higher than DEHPh.^18−20^ DEHA was also reported to induce damaging effects on the brain, heart, and liver tissues due to oxidative stress, inflammation, and apoptosis,^21^ while exposure to diethylene glycol dibenzoate (DEGDB) could increase lipid production and mobilization in a nonmonotonic pattern.^22^ Several other commonly used NPPs, such as ATBC, TOTM, and TMPDDiB, have also been reported to cause endocrine/reproductive toxicity, organ/tissue destruction, or cytotoxicity.^17,23−27^ Elucidation of their relative environmental and human health risks to PAEs requires not only better investigations of toxicological potencies but also improved knowledge on the environmental distributions and abundances.

Humans spend most of the time indoors.^28^ The indoor environment, particularly the home environment, has been suggested as one of the important microenvironments where humans are exposed to industrial chemicals.^29−31^ Indoor dust has been demonstrated by numerous studies as a convenient and efficient proxy for evaluating indoor exposure to industrial chemicals.^32^ Indeed, previous studies have reported ubiquitous distributions of PAEs in house dust from different countries and regions, with levels ranging up to 2400 μg/g.^33−37^ Studies also reported the associations between house dust concentrations of PAEs and the levels in human urine or hair, demonstrating the importance of indoor exposure to human health risks.^38−40^

Although recent studies have increasingly reported the occurrences of NPPs in the indoor environment, available data have generally been focused on a few selected ones, such as ATBC, DEHA, DEHT, DINCH, and TOTM.^36,41,42^ Information on other emerging NPPs remains very limited not just in the indoor environment but also in other environmental compartments. Given the potential toxicity of NPPs and their increasing applications in household consumer products, understanding of their indoor contamination status and potential human exposure risks becomes a critical need. Therefore, in the present study, we focused on a suite of 45 nonphthalate plasticizers to explore their abundances and profiles in house dust from multiple locations in the Asia-Pacific region and the United States (U.S.). PAEs were also determined in order to achieve a full understanding of plasticizer contamination in the house environment from different regions. Specific objectives of this work were to (1) characterize plasticizer contamination profiles in house dust and understand region-specific contamination status and characteristics and (2) estimate human exposure risks to the nonphthalate plasticizers via dust ingestion and dermal contact. The novelty of our work stems from the uncovering of a suite of emerging NPPs in multiple regions across different continents, demonstrating their broad occurrences in the home environment and raising concerns on the related human exposure and health risks. This is noteworthy as a portion of the emerging NPPs had rarely been concerned by previous environmental investigations.

## Materials and Methods

### Chemical and Reagents

**Figure 1:**
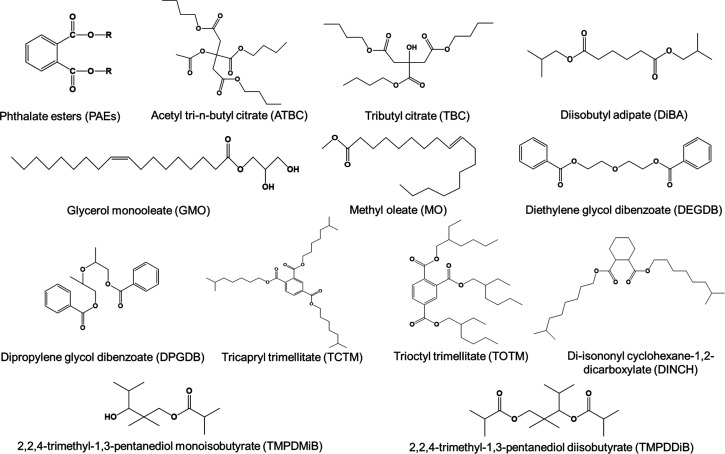
Chemical structures for phthalate esters and major nonphthalate plasticizers.

Reference standards of 45 nonphthalate plasticizers and 24 phthalates (Table S2 and Figure 1) were purchased from AccuStandard (New Haven, CT). Thirteen isotopically labeled standards were used as surrogate standards, while coumaphos-d10 and *tert*-butyl paraben-d9 were employed as internal standards (Table S2). They were obtained from AccuStandard, Toronto Research Chemicals, or Wellington Laboratories (Guelph, ON).

### Sample Collection

Sampling protocols have been introduced in our earlier publications.^29−31^ In brief, residential house dust was collected during the period of August 2017 to May 2022 from five different regions, including the city of Tianjin, North China (*n* = 36 homes), city of Guangzhou, South China (*n* = 41), city of Adelaide, South Australia (*n* = 42), city of Carbondale, Illinois (U.S.; *n* = 17), and city of Hanoi, Vietnam (*n* = 21). A customized nylon bag (pore size = ∼25 μm) was attached to the floor attachment of a commercial vacuum cleaner. After vacuuming the floors, the nylon bag was detached and wrapped with a clean aluminum foil. Precleaned sodium sulfate was used as field blanks, which was vacuumed and prepared in the same way as for dust collection. A field blank was prepared for every five homes. In addition, indoor temperature and humidity were also recorded by investigators during home visits. Dust and sodium sulfate were removed from the nylon bag, and it was sieved through a 125 μm stainless cloth sieve (Hogentogler & Co., Inc., Columbia, MD, U.S.) and stored at −20 °C prior to chemical analysis.

### Sample Analysis

Approximately 20–50 mg of sieved dust was spiked with a mixture of surrogate standards and extracted with 3 mL of acetonitrile under sonication (20 min). The mixture was centrifuged at 3500 rpm for 5 min, and the supernatant was transferred to a glass tube. The extraction was repeated twice, and the extracts were combined, concentrated under gentle nitrogen flow, and filtered through a centrifugal filter (VWR International, 0.22 μm). The final extract was reconstituted with methanol and spiked with internal standards prior to instrumental analysis.

Nonphthalate and phthalate plasticizers were determined using an ultraperformance liquid chromatograph (UPLC) coupled to a 5500 Q Trap triple quadrupole mass spectrometer and operated in the positive electrospray ionization (ESI) mode (AB Sciex, Toronto, Canada). The UPLC was equipped with a Luna 2.5 μm C18(2) 100 Å column (100 mm × 2 mm, 3 μm particle size; Phenomenex, Torrance, CA, U.S.). Di-*n*-butyl maleate and dibutyl fumarate were coeluted, and their concentrations were determined together as DBM/DBF. Similarly, diisooctyl azelate (DiOAZ) and di(2-ethylhexyl) azelate (DEHAZ) were also reported together due to coelution. Detailed information about the instrumental analysis and chemical-dependent instrumental parameters is summarized in the Supporting Information (SI).

### Quality Assurance and Quality Control

Other than field blanks, a laboratory procedural blank was also processed along with each batch of 10 authentic samples to evaluate background contamination. Trace amounts of glycerol monooleate (GMO), methyl oleate (MO), tributyl citrate (TBC), tricapryl trimellitate (TCTM), dipropylene glycol dibenzoate (DPGDB), TMPDDiB, and isopropyl palmitate (IPP) were detected in procedural and field blanks, with an average mass of 3.54–43.1 ng. Eight PAEs, including dimethyl phthalate (DMPh), diethyl phthalate (DEPh), dibutyl phthalate (DBPh), diisobutyl phthalate (DiBPh), DEHPh, diphenyl phthalate (DPPh), and butyl benzyl phthalate (BBzPh), were also detected in procedural and field blanks, accounting for 0.02–2.6% of the median levels detected in dust samples. Reported concentrations of these chemicals were corrected with blank contamination and the recoveries of their corresponding surrogate standards. The limit of quantification (LOQ) of an analyte with background contamination was defined as the average contamination levels in the blanks plus 10 times the standard deviation of the background contamination;^43^ otherwise, the LOQ was determined as the instrumental response 10 times the standard deviation of the noise. The LOQs of target analytes ranged from 1.5 to 75 ng/g for NPPs and 4 to 110 ng/g for PAEs. More details are summarized in Table S2.

Extraction efficiencies were evaluated via matrix spiking tests, where approximately 50 mg of a pooled dust sample was spiked with target and surrogate standards and processed with the aforementioned method, along with two matrix blanks (only addition of surrogate standards). After subtracting the levels in matrix blanks, the recoveries of target chemicals from analytical procedures ranged from 51 ± 15% to 136 ± 14% (Table S2). Matrix effects were evaluated for these plasticizers following the method described in the previous study^44^ and summarized in the SI. The determined matrix effects ranged from 74 ± 6% to 127 ± 24% for NPPs and 79 ± 16% to 147 ± 34% for PAEs (Table S2). Recoveries of surrogate standards during the analysis of authentic samples ranged from 75 ± 16% to 139 ± 25%.

### Exposure Assessment and Data Analysis

Daily intake (EDI) of plasticizers via dust ingestion or dermal contact was determined using the following equations^29,45^
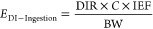
1
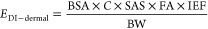
2where *E*_DI_ is the estimated daily intake (ng/kg body weight/day), *C* is the concentration of a chemical in house dust (ng/g), IEF is the indoor exposure fraction (hours spent over a day in homes), DIR is the dust ingestion rate (g/day), BW is body weight (kg), BSA is body surface area (cm^2^/day), SAS is the amount of solid particles adhered onto skin (mg/cm^2^), and FA is the fraction of a chemical absorbed through the skin. We assumed a 100% absorption of chemicals from ingested dust. Due to the lack of experimental and model data of skin absorption of NPPs, the skin absorption fraction of NPPs was assumed to be 0.000031 (low exposure) or 0.01025 (high exposure) according to the experimental data of PAEs (0.000031–0.01025).^46^ Other parameters included in the equations are summarized in Table S3.

The hazard quotient (HQ) was determined to assess human exposure risks via dust ingestion and dermal absorption. Only chemicals with a DF of >70% in at least four of the five regions were included for HQ estimation^47^
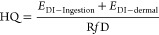
3where R*f*D represents the reference dose of a target chemical. For an analyte without an appropriate R*f*D, its nonobserved-adverse-effect-level (NOAEL) or lethal dose (LD_50_) adjusted with an uncertainty factor was applied (Table S4). A hazard index (HI) was also calculated by summing the HQs for individual analytes.

For a target analyte with a detection frequency (DF) > 70%, an LOQ/√2 was assigned to any measurements below the LOQ for statistical analysis. Statistical analyses and data visualization were conducted using Origin version 9.0 or PASW Statistics 18.0. Differences among chemical groups or regions were determined using a Kruskal–Wallis analyses of variance (ANOVA) followed by a Mann–Whitney test. Spearman’s correlation analyses were used to determine the relationships between individual plasticizers in house dust. The level of significance was set at α = 0.05.

## Results and Discussion

### Profiles of NPPs in House Dust

**Figure 2:**
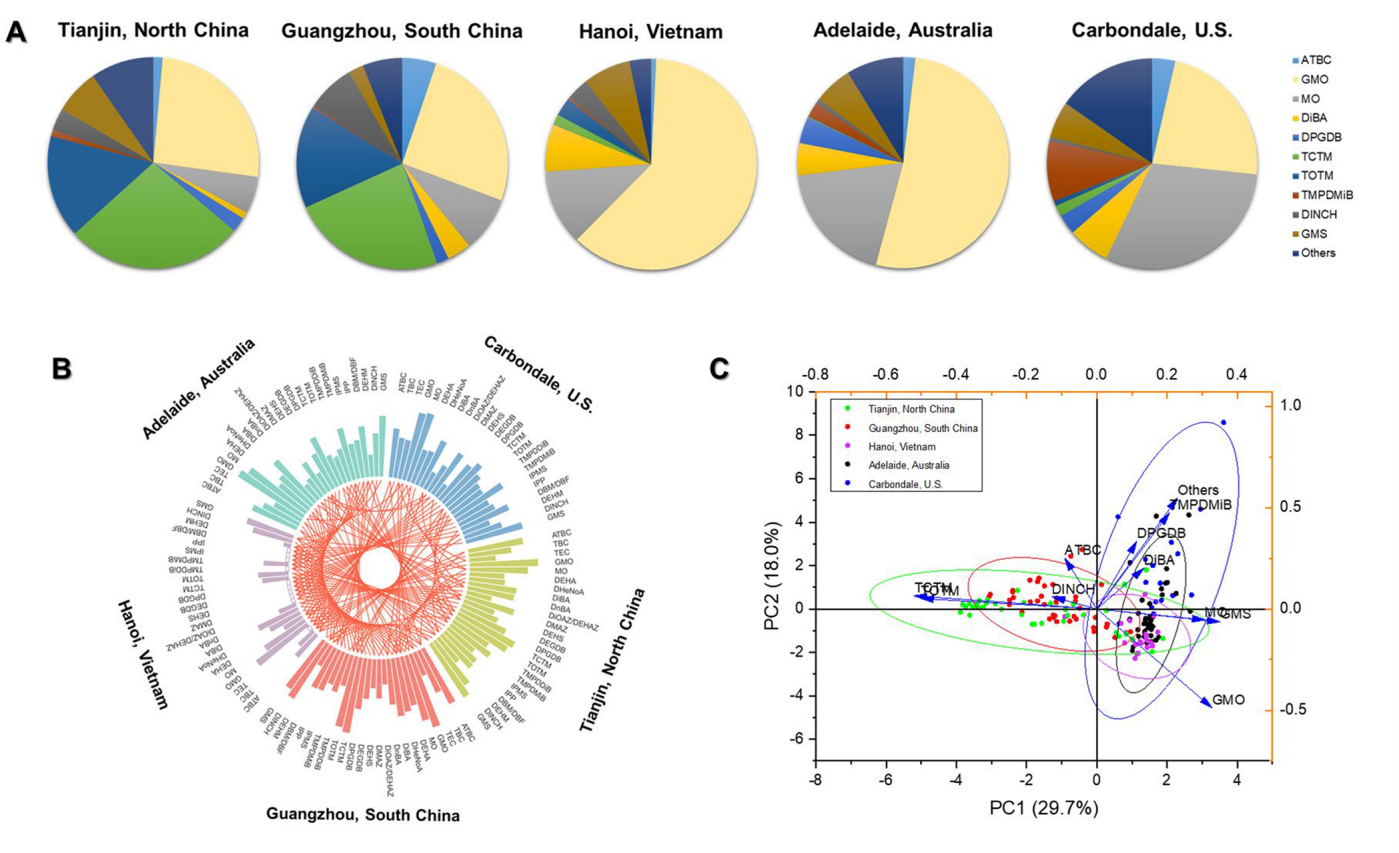
(A) Chemical compositions of major nonphthalate plasticizers in indoor dust from different regions; (B) comparison of concentration for nonphthalate plasticizers from different regions (The columns represent the median concentrations, and the red lines represent significant differences in compounds between the two regions.); and (C) principal component analysis of major nonphthalate plasticizers in indoor dust from different regions.

Among the 45 nonphthalate plasticizers, ATBC, DEHA, diisobutyl adipate (DiBA), DINCH, GMO, MO, TCTM, and TOTM exhibited a detection frequency of 100% in house dust from the five studied locations, while 16 other chemicals (e.g., DEGDB, DEHA, di(2-ethylhexyl) maleate (DEHM), DPGDB, glycerol monostearate (GMS), and TBC) were detected in more than 70% of house dust from at least four of the five locations (Table 1, Figure 2A). This indicates a widespread distribution of the variety of NPPs in the house environment from different countries. Several NPPs, including *n*-butyl acetyl ricinoleate (BARO), methyl o-acetylricinoleate (MARO), and tetrahydrofurfuryl oleate (THFO), were not found in any location. Combined together, the total concentrations of NPPs exhibited a median value of 199 μg/g in Tianjin (North China), 252 μg/g in Guangzhou (South China), 17.8 μg/g in Hanoi (Vietnam), 115 μg/g in Adelaide (Australia), and 116 μg/g in Carbondale (U.S.).

**Table 1:** Concentrations (μg/g) of Nonphthalate Plasticizers in House Dust from Multiple Countries^a^^,^^b^

	Tianjin, North China			Guangzhou, South China			Hanoi, Vietnam			Adelaide, Australia			Carbondale, U.S.		
	DF	median	range	DF	median	range	DF	median	range	DF	median	range	DF	median	range
ATBC	100	3.12	0.05–20.1	100	7.21	2.23–62.5	100	0.16	0.04–0.64	100	2.43	0.34–21.7	100	1.17	0.53–7.61
BTHC	83	0.004	nd-0.24	37	nd	nd-0.17	71	0.005	nd-0.02	31	nd	nd-0.03	100	0.03	0.004–0.31
TBC	94	0.50	0.007–4.48	100	0.54	0.06–7.22	62	0.02	nd-0.41	100	0.35	0.03–2.88	100	0.36	0.07–2.57
TEC	94	0.05	nd-10.2	95	0.10	nd-0.67	24	nd	nd-1.20	100	0.10	0.01–1.18	100	0.31	0.03–10.2
BO	94	1.18	nd-24.8	100	0.47	0.11–4.37	5	nd	nd-0.23	39	nd	nd-1.03	82	0.31	nd-2.60
BRO	39	nd	nd-0.33	88	0.23	nd-1.71	86	0.04	nd-0.13	39	nd	nd-0.79	24	nd	nd-0.23
GMO	100	4.91	0.27–175	100	57.8	10.6–265	100	9.59	1.08–1.85	100	67.4	1.43–545	100	20.8	1.92–88.5
MO	97	4.56	nd-77.7	100	14.6	2.88–94.7	100	1.13	0.13–16.0	100	26.2	0.65–287	100	27.8	3.35–93.1
PO	86	0.18	nd-1.96	56	0.10	nd-0.61	0	nd	nd	81	0.18	nd-3.68	71	0.26	nd-1.63
DEHA	100	0.16	0.02–1.37	100	1.41	0.58–13.1	100	0.05	0.01–1.05	100	0.50	0.04–11.0	100	0.17	0.06–1.58
DHeNoA	92	0.19	nd-11.5	96	2.82	nd-8.14	95	0.31	nd-1.38	98	0.99	nd-5.84	77	0.41	nd-0.97
DiBA	100	1.12	0.13–18.4	100	3.72	1.01–124	100	1.22	0.07–63.1	100	7.58	0.38–95.5	100	3.17	1.35–43.8
DiDeA	100	0.78	0.10–10.4	98	0.57	nd-1.32	10	nd	nd-0.016	61	0.02	nd-0.34	35	nd	nd-0.15
DMA	100	1.60	0.22–18.5	20	nd	nd-1.54	0	nd	nd	20	nd	nd-5.12	88	0.32	nd-32.9
DnBA	100	0.94	0.09–12.8	100	0.68	0.17–15.1	91	0.03	nd-0.38	98	0.04	nd-0.75	100	0.02	nd-2.67
DiOAZ/DEHAZ	86	0.04	nd-0.34	94	0.03	nd-0.51	33	nd	nd-0.10	76	0.03	nd-0.19	100	0.08	0.01–1.02
DMAZ	94	0.21	nd-6.44	100	0.24	0.05–8.72	0	nd	nd	88	0.23	nd-34.4	94	1.26	nd-21.7
DEHS	100	0.11	0.01–1.68	84	0.08	nd-0.57	38	nd	nd-0.03	95	0.02	nd-0.60	82	0.01	nd-3.21
DMS	75	0.09	nd-0.56	84	0.05	nd-2.44	0	nd	nd	12	nd	nd-0.54	82	0.03	nd-0.38
DEGDB	97	0.28	nd-10.1	100	0.41	0.11–8.23	19	nd	nd-0.03	100	0.31	0.02–5.49	100	0.73	0.07–10.1
DPGDB	97	1.48	nd-32.7	100	1.51	0.27–67.1	43	nd	nd-0.06	100	2.57	0.26–34.2	100	2.21	0.24–9.45
TCTM	100	10.2	0.16–630	100	50.7	3.71–297	100	0.11	0.01–6.61	100	0.20	0.01–1.59	100	0.65	0.11–8.48
THTM	81	0.002	nd-0.07	54	0.004	nd-0.02	0	nd	nd	37	nd	nd-0.02	71	0.01	nd-0.24
TiNTM	97	0.04	nd-0.47	88	0.06	nd-4.71	29	nd	nd-0.30	42	nd	nd-0.09	71	0.04	nd-0.47
TOTM	100	5.79	0.08–560	100	29.7	3.66–389	100	0.17	0.02–14.0	100	0.12	0.007–0.53	100	0.27	0.06–4.33
TMPDDiB	100	0.46	0.03–5.72	95	0.13	nd-0.88	38	nd	nd-0.09	90	0.14	nd-5.54	100	0.23	0.07–4.27
TMPDMiB	100	1.29	0.20–8.53	95	0.39	nd-2.96	57	0.04	nd-0.61	98	2.16	nd-8.23	100	3.94	0.67–75.2
IPMS	94	0.15	nd-3.86	88	0.14	nd-2.60	5	nd	nd-0.05	100	0.18	0.05–28.3	94	0.21	nd-10.7
IPP	89	0.48	nd-6.22	100	0.63	0.10–4.64	5	nd	nd-0.04	98	0.25	nd-5.46	100	0.33	0.04–3.82
DBM/DBF	100	0.55	0.12–4.35	98	0.71	nd-6.27	10	nd	nd-0.03	100	1.57	0.1–34.8	100	1.88	0.18–6.67
DEHM	100	0.09	0.01–1.45	100	0.04	0.009–0.24	62	0.003	nd-0.03	100	0.03	0.005–1.28	100	0.07	0.02–1.36
DINCH	89	2.50	nd-26.7	100	11.5	4.61–307	100	0.77	0.08–4.60	100	1.01	0.12–16.1	100	0.30	0.08–1.50
GMS	100	9.45	0.77–51.6	90	6.11	nd-31.6	100	1.26	0.02–8.86	100	6.02	0.94–68.2	100	4.79	0.88–14.5
Σ45NPPs		199	10.0–1310		252	94.3–922		17.8	1.55–203		115	26.9–748		116	25.6–222
Σ24PAEs		496	132–1880		520	211–1720		153	73–825		295	42–747		480	254–1410

a Only chemicals with a detection frequency > 50% in at least one region are summarized in this table.

b ATBC: acetyl tri-*n*-butyl citrate; BTHC: n-butyryltri-*n*-hexyl citrate; TBC: tributyl citrate; TEC: triethyl citrate; BO: butyl oleate; BRO: butyl ricinoleate; GMO: glycerol monooleate; MO: methyl oleate; PO: n-propyl oleate; DEHA: bis(2-ethylhexyl) adipate; DHeNoA: di(n-heptyl,n-nonyl) adipate; DiBA: diisobutyl adipate; DiDeA: diisodecyl adipate; DMA: dimethyl adipate; DnBA: dibutyl adipate; DiOAZ/DEHAZ: diisooctyl azelate/di(2-ethylhexyl) azelate; DMAZ: dimethyl azelate; DEHS: di(2-ethylhexyl) sebacate; DMS: dimethyl sebacate; DEGDB: diethylene glycol dibenzoate; DPGDB: dipropylene glycol dibenzoate; TCTM: tricapryl trimellitate; THTM: trihexyl trimellitate; TiNTM: triisononyl trimellitate; TOTM: trioctyl trimellitate; TMPDDiB: 2,2,4-trimethyl-1,3-pentanediol diisobutyrate; TMPDMiB: 2,2,4-trimethyl-1,3-pentanediol monoisobutyrate; IPMS: isopropyl myristate; IPP: isopropyl palmitate; DBM/DBF: di-*n*-butyl maleate/dibutyl fumarate; DEHM: di(2-ethylhexyl) maleate; DINCH: di(2-ethylhexyl) maleate; GMS: glycerol monostearate.

**Figure 3:**
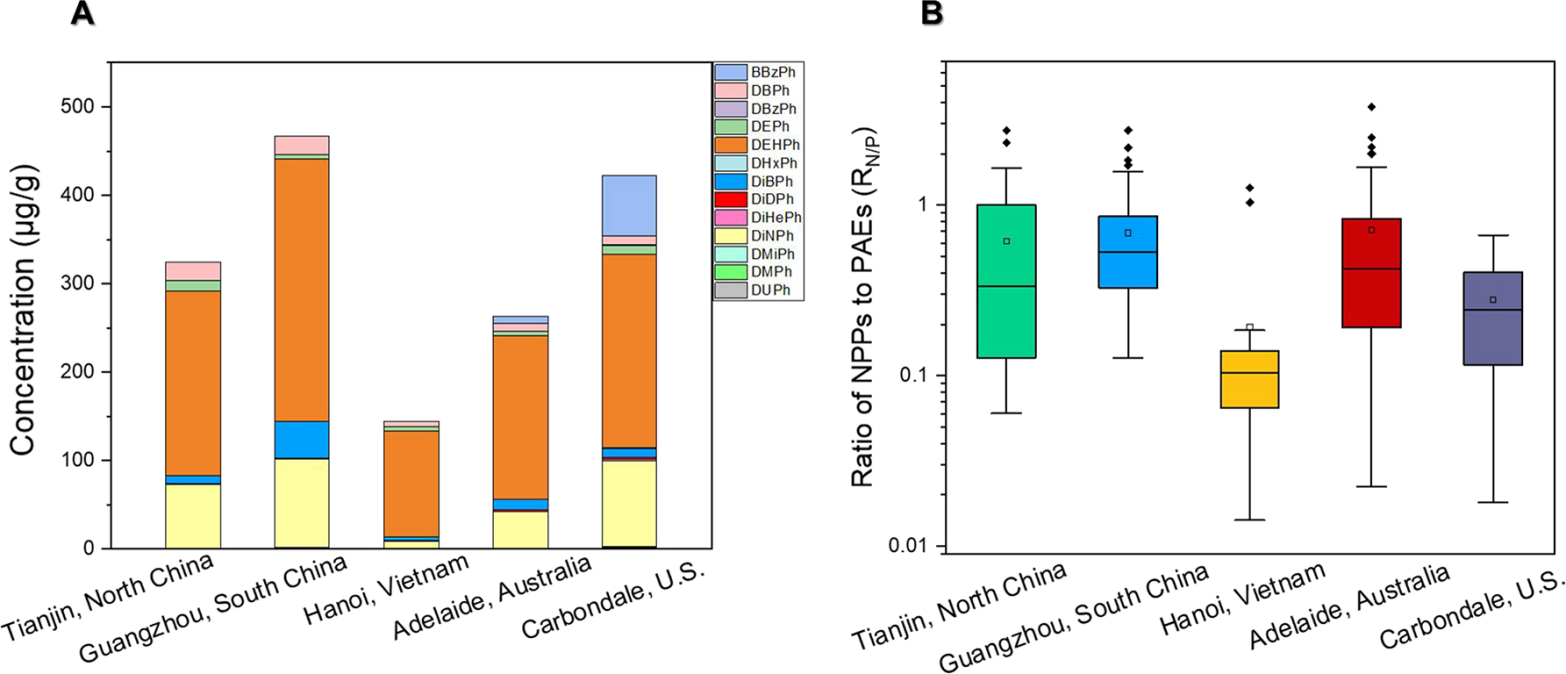
(A) Median concentrations (μg/g) of major phthalates in indoor dust from different regions and (B) total concentration ratios of nonphthalate plasticizers and phthalates in indoor dust from different regions.

To better understand the contamination scenarios of NPPs in the indoor environment, we also determined the major PAEs in the same dust for comparison. The median levels of ΣPAEs were determined to be 496, 520, 153, 295, and 480 μg/g in house dust from Tianjin, Guangzhou, Hanoi, Adelaide, and Carbondale, respectively (Table 1, Figure 3A, and Table S5). The mean ratios of ΣNPPs to ΣPAEs ranged from 0.19 (Hanoi) to 0.72 (Adelaide) in these five locations (Figure 3B). The production volume ratio of NPPs to PAEs reported in Global Consumption of Plasticizers (0.54)^3^ was much lower than the ΣNPPs/ΣPAEs ratio determined in house dust from Tianjin (0.61), Guangzhou (0.68), and Adelaide (0.72), indicating that NPPs could have been massively used in consumer products in these two countries. By contrast, the low ΣNPPs/ΣPAEs values in dust from Hanoi may indicate that PAEs are still dominant in the local plasticizer market. Regardless, it merits attention that in the five locations the concentrations of several NPPs (e.g., GMO, MO, DPGDB, DiBA, and TCTM) were consistently comparable to or higher than those of individual PAEs except for DEHP in the same dust (Table 1, Figure 3A, and Table S5).

Among the top 10 NPPs detected in house dust from the study locations (Figure 2A), a few of them have rarely been investigated for their environmental occurrences and distributions. They mainly included GMO, MO, TCTM, GMS, TMPDMiB, and IPP. GMO is mainly used as an additive in the formation of liquid crystalline drug formulations, friction modifier, and as a processing aid in the production of emulsions and foams in food.^48−52^ The long use history and extensive applications in consumer products and food may lead to high indoor contamination. Another oleate-based plasticizer MO, which was also abundant in house dust, finds applications in biofuel, agrochemicals, lubricants, cleaners, metal working fluids, oiling agent for textiles, solvents, and personal care products.^53^

TCTM, as well as several other citrate-based plasticizers (i.e., ATBC, TBC, and triethyl citrate (TEC)), exhibited broad distributions and high concentrations in house dust. These chemicals are primarily used as a monomer for functional or biodegradable polymers and as a water softener or a complexing agent in detergents or metal treatment, respectively.^54^ Previous studies have indicated that some citrates may migrate from consumer products, thus causing widespread contamination in the indoor environment. Indeed, several recent studies have reported the presence of ATBC, TBC, and TEC in European and Chinese house dust, as well as in food and river water.^36,55−58^ However, TCTM had not been reported by any environmental studies prior to our work.

TMPDMiB and TMPDDiB exhibited broad occurrences in house dust, except in that from Hanoi. They are mainly used as a coalescing aid in latex paints and other products,^3^ and both are listed as high production volume (HPV) chemicals according to the US Environmental Protection Agency (EPA) HPV Information System.^59^ However, environmental studies on these two butyrate-based plasticizers are very scarce. Addington et al. (2020) reported their migration from household articles, which likely contributes to their broad occurrences in the home environment.^2^

Other than the top 10 abundant NPPs, a few adipate-, azelate-, fumarate-, maleate-, myristate-, and sebacate-based chemicals, such as DBM/DBF, DEHA, di(2-ethylhexyl) sebacate (DEHS), DiOAZ/DEHAZ, DiBA, dibutyl adipate (DnBA), and dimethyl azelate (DMAZ), exhibited high detection frequencies in most of the study regions, although their concentrations were generally 1–3 orders of magnitude lower than the top abundant NPPs. These plasticizers are mainly used as additives in food-contact materials, personal care products, fabrics, and textiles, as well as in a variety of other consumer products (US EPA’s CHEMVIEW database).^60^ Available environmental studies on some of these plasticizers (i.e., DiBA, DnBA, and DMAZ) are mainly limited in the indoor environment, but knowledge on their distributions in other environmental compartments is limited.

Overall, our data clearly indicate the ubiquitous distributions of a number of NPPs in the indoor environment. The restrictions in the production and applications of PAEs have consequently stimulated the increasing use of the NPPs to replace PAEs in order to meet industrial needs. While these chemicals may have emerged as one of the most abundant indoor chemicals, our knowledge on their environmental distributions, sources, and fate remains very limited for most of these NPPs. This raises the critical need of continued monitoring of their distributions and behavior in various environmental compartments, including but not limited to the indoor environment.

### Spatial Variations of NPPs Contamination

The ΣNPPs concentrations exhibited significant differences among the five study locations (Table 1). In particular, both North and South China contained significantly higher concentrations than any of the other three locations (*p* < 0.05), while the NPP concentrations in Hanoi were significantly lower than that of any of the other four regions (*p* < 0.05; Table 1 and Figure 2B). A similar pattern was also observed for PAEs, further demonstrating the heavy applications of plasticizers in China’s consumer products (Figure 3A and Table S5). This is in line with the proportion of countries/regions in Global Consumption of Plasticizers (2017), with China accounting for the highest proportion (42%), followed by the Western Europe (14%) and the U.S. (11%).^3^

In addition to the regional differences in concentration levels, the compositional profiles of NPPs also exhibited unlike patterns across locations (Figure 2A). Among the top ten NPPs which constituted an average of 85% to 97% of total NPPs in house dust, GMO and TCTM appeared to be the most abundant NPPs in both North China and South China house dust, followed by TOTM. In addition, MO, DINCH, ATBC, and DiBA, but not GMS, also have similar abundance orders between the two locations. Although GMO remained the most abundant NPP in both Australia and Hanoi dust samples, decreased compositions were observed for TCTM and TOTM compared with those in China dust. By contrast, in Carbondale house dust, MO appeared to be the most abundant, followed by GMO, GMS, TMPDMiB, DiBA, and others. Indeed, the PCA analysis of the compositions of major NPPs indicated large spatial differences, particularly between the data from China and other locations (Figure 2C). By contrast, the profiles of PAEs exhibited less variance among the five regions (Figure 3A). They were all dominated by DEHP, followed by DiNP, DiBP or DBP or BBzP, although some differences in compositions occurred for selected chemicals.

Therefore, our data indicate region-specific market demand and application patterns of the NPPs. It should also be noted that the spatial variations may be confounded by different sampling times and inconsistency in the representativeness of the study locations for the entire region. For example, Carbondale is a relatively small university town and may not be representative of the entire mid-Western U.S., while the other four cities as metropolitan centers are more representative of each corresponding region. Nevertheless, given that different NPPs may differ in toxic effects and health risks, the region-specific contamination pattern should be taken into consideration when assessing NPP contamination and exposure risks from a regional or global perspective.

### Human Exposure Assessment

The risks of human exposure to NPPs were estimated by evaluating two exposure routes: dust ingestion and dermal contact. The median daily intakes of NPPs via dust ingestion by toddlers and adults from the five regions were estimated to be 74.9–1060 ng/kg bw/day and 3.83–54.2 ng/kg bw/day, respectively, under the average exposure scenarios, and 300–4240 ng/kg bw/day and 9.57–136 ng/kg bw/day, respectively, under the high exposure scenarios (Table S6). However, even under the high exposure scenarios, the median EDIs of NPPs via dermal sorption were determined to be 3.78–53.5 ng/kg bw/day and 0.87–12.3 ng/kg bw/day for toddlers and adults, respectively, generally lower than the estimations via dust ingestion (Table S6). Toddlers appeared to be subjected to elevated exposure compared with adults because of their lower body weights, higher dust ingestion rates, and more time spent indoors than adults.^29^

**Figure 4:**
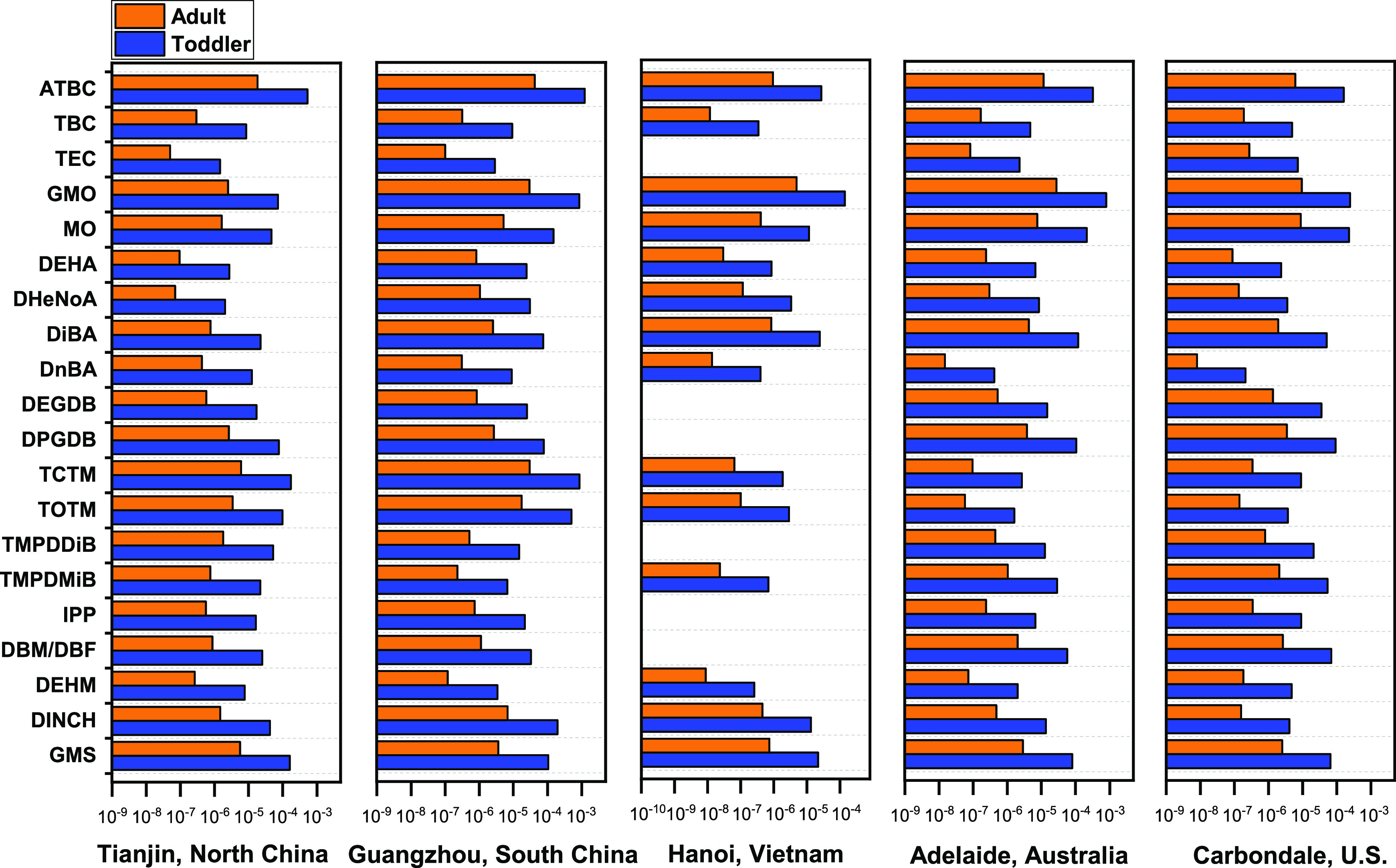
Median HQs of individual nonphthalate plasticizers by adults and children via dust ingestion and dermal contact. Only chemicals with a detection frequency > 90% in at least four regions are described in this figure.

The HQs of individual NPPs, as well as the HI, were determined to be less than 1 for both toddlers and adults in the five regions even under the highest exposure scenarios (Figure 4, Table S7). This suggests that the intake through dust ingestion and dermal contact unlikely caused substantial human health risks at the current exposure levels. However, the HQ prioritization of individual chemicals exhibited a region-specific pattern. GMO exhibited the highest HQ in Hanoi, Adelaide, and Carbondale but not in Tianjin and Guangzhou where ATBC contributed the most to the HQ, followed by TCTM or GMO. This indicates a region-specific exposure risk not just because of the differences in chemical abundances but also due to chemical-specific toxic effects. Despite limited toxicological evaluations on GMO, Fang et al. reported that dust-associated oleic acids and myristic acids could contribute substantially to the human peroxisome proliferator-activated nuclear receptors (PPARγ 1) activity in house dust.^61^

Despite low exposure risks according to the HQ estimation, cautions are needed when interpreting the above findings. First, as the majority of NPPs lack corresponding R*f*Ds or other toxic thresholds, their HQs were estimated based on the predicted R*f*D values from the LD_50_ or NOAEL for various end points. This may result in large deviations from accurate determination of the actual health risks. Second, the above HI approach combines the HQs from individual chemicals but ignores synergistic or antagonistic effects caused by coexisting chemicals, as well as their diverse toxic modes of action and thresholds. For example, binary exposure to DBPh and DEHPh could synergistically induce hypospadias in 43.3% of male rates, while hypospadias was only observed in 0% and 1.9% of rats treated with DBPh and DEHPh, respectively.^62^ Additionally, glycerin monostearate, a chemical structurally similar to GMO, has been reported to enhance the toxicity of DEHPh on male reproduction.^63^ Third, additional exposure routes (e.g., inhalation and dietary intake) other than dust ingestion and dermal contact may also contribute to human exposure to the plasticizers. Indeed, DINCH, DEHA, and DiBA are widely employed in the manufacturing of food packaging products,^64,65^ which contact with food subsequently and enhance the opportunity of exposure to these chemicals via food intake. In addition, ATBC and GMO have been reported to be directly used in the food industry as food additives.^48−52^ Thus, food intake could represent an important exposure pathway for selected plasticizers. These factors demonstrate that we need a better understanding of chemical-specific exposure pathways, toxicokinetics, and modes of action in order to achieve an accurate estimation of the exposure risks to the variety of plasticizers.

## Environmental Implications

As one of the very few environmental investigations on the emerging plasticizers, our multicountry study clearly demonstrated the broad occurrences and region-specific contamination profiles of a large variety of NPPs in the indoor environment. Despite that many NPPs have been reported in previous studies, existing knowledge on their environmental occurrences and distributions remains still limited in both the number of reports and geographical ranges. Current data are apparently insufficient to support risk assessments, which raises the urgent need for better characterization of their distributions, fate, and health risks, especially across different regions. Our findings are expected to attract more attention from scientific communities on these emerging chemicals and facilitate related environmental investigations and risk assessments. Our work also presents a couple of important implications as emphasized below.

Whether the variety of NPPs represents higher or lower risks than PAEs requires better elucidations. It seems unlikely to reach any definitive conclusion on whether the NPPs are safe replacements or just regrettable substitutes, due to much limited knowledge on the environmental and toxicological data of NPPs, which is in sharp contrast with numerous PAE studies. Existing environmental monitoring mostly focused on a limited number of well-known PAE replacements (e.g., DINCH), but many emerging plasticizers have not been covered. It is clear that the various groups of emerging NPPs differ in chemical structures, raising the need of evaluating their chemical-specific environmental behavior, fate, and distributions among different environmental compartments or across different geographical territories (particularly remote areas). In addition, knowledge on the toxicological profiles of most NPPs lags even further behind. This limits efficient assessment of the risks of NPP exposure and in particular their relative risks to PAEs. Therefore, comparative toxicity evaluations between NPPs and PAEs are critically needed.

Attention should be given to an increasing list of plasticizers. Other than the NPPs included in our work, additional novel NPPs may also exist. Even for the known target NPPs, there may be additional congeners or structurally similar chemicals undergoing industrial applications or existing as impurities. Environmental or biological transformation could also produce a suite of structurally related products with environmental relevance. Indeed, nontarget and suspect screening has identified a number of new citrate chemicals as well as many other “novel” additive chemicals in the indoor environment and foodstuff.^36,55,56,66^ The increasing list of novel NPPs and related chemicals would substantially increase the environmental risks of emerging plasticizers.

Human exposure risks to emerging plasticizers require better elucidations. This needs better characterization of chemical-specific exposure pathways and appropriate exposure markers. As we discussed earlier, the plasticizers may differ greatly in their dominant exposure pathways due to specific applications and unlike physicochemical properties. This subsequently results in chemical-specific and gender or age-specific exposure risks. In addition, given most of the plasticizers may be subjected to biological transformation, their dominant transformation products instead of plasticizers themselves could be appropriate exposure markers for biomonitoring investigations. However, the exposure markers have not been confirmed for most of the NPPs to date. More importantly, efforts are critically needed to elucidate the cocktail effects in humans from the simultaneous exposure to a variety of NPPs along with the PAEs.

Overall, although the PAEs remain dominant in the indoor environment from the study regions, continuous and large-scale applications of NPPs may result in increasing contamination along with time, raising concerns on the subsequent human exposure risks. While the current exposure may unlikely cause significant health risks according to the hazard quotient estimation, the underestimation of exposure risks cannot be excluded due to the lack of appropriate toxic threshold data, the existence of additional exposure pathways, and possible cocktail effects from coexisting NPPs and PAEs. Thus, continuing efforts are needed to monitor the environmental distributions and fate of NPPs and characterize the related human exposure risks.

## Supporting Information Available

Detailed description of sample analysis and matrix effect evaluation; instrumental analysis parameters (Table S1); summary of analytes and their key instrumental parameters and QA/QC results (Table S2); parameters for daily intake estimation (Table S3); reference doses and hazard quotients (Table S4); concentrations of selected analytes (Table S5); estimated daily intakes (Table S6); median hazard quotients of nonphthalate plasticizers (Table S7) (PDF)
