# Supplementary material for: Nonphthalate Plasticizers in House Dust from Multiple Countries: An Increasing Threat to Humans
Source: Environ Sci Technol. 2023 Feb 22;57(9):3634–44. doi: 10.1021/acs.est.2c08110 (PMC9996830; doi:10.1021/acs.est.2c08110)
Supplement: Supplementary file 1 — es2c08110_si_001.pdf [file es2c08110_si_001.pdf]

Nonphthalate Plasticizers in House Dust from Multiple Countries: An Increasing Threat to Humans?

Hongli Tan[1], Liu Yang[2], Xiaolin Liang[2], Diedie Huang[2], Xinhang Qiao[2], Qingyuan Dai[1], Da Chen[2*], Zongwei Cai[1*]

[1] State Key Laboratory of Environmental and Biological Analysis, Hong Kong Baptist University, Hong Kong SAR, 999077, China

[2] School of Environment, Guangdong Key Laboratory of Environmental Pollution and Health, Jinan University, Guangzhou, 510632, China

Number of pages: 15

Number of figures: 0

Number of table: 7

***Corresponding author: Zongwei Cai,** E-mail: zwcai@hkbu.edu.hk; **Da Chen**, E-mail: dachen@jnu.edu.cn

Method for the determination of phthalate esters and nonphthalate plasticizers

Determination of phthalate esters and nonphthalate plasticizers was conducted on an ultra-performance liquid chromatograph coupled to a 5500 Q Trap triple quadrupole mass spectrometry (AB Sciex, Toronto, Canada). The liquid chromatograph was equipped with a Luna® 3 µm C18(2) 100 Å column (100 mm × 2 mm, 3 µm particle size; Phenomenex, Torrance, CA, U.S.). The mobile phases and gradients for the analysis of each group of chemicals were summarized in **Table S1**. The mass spectrometry was operated in the multiple reaction monitoring (MRM) mode with a positive or negative electrospray ionization (ESI) mode. The MRM transitions and other chemical-dependent instrumental parameters are summarized in **Table S2**.

Matrix effect evaluation

Matrix effects were evaluated with the method described in Xie et al. (2021).[1] Six replicates of an in-house dust composite (100 mg each) was extracted without addition of any target analytes or surrogate standards. The final extract was reconstituted in 100 µL of methanol and then aliquoted into two sub-samples with equal volume (50 µL each). Sub-sample A was spiked with 50 µL of a standard mixture of analytes (50 ng/mL per analyte). Sub-sample B was spiked with 50 µL of methanol. An external standard solution (S) was prepared by mixing the 50 µL of analyte mixtures (50 ng/mL each) with 50 µL methanol. By comparing the response differences of the analytes in the sub-samples A and B to the responses of the analytes in the external standard, a matrix effect (ME) value was calculated as:

$$ME(\%) = 100 \times \frac{(Ai - Bi)}{Si}$$

where Ai, Bi and Si are the chromatographic peak areas of the analyte (i) in sub-samples A and B and external standard solution (S), respectively. The matrix effect results are shown in **Table S2**.

Table S1. Information on the chromatographic columns, parameters, and gradient programs of UPLC-MS/MS analysis for target chemicals.

Column	Flow Rate (mL/min)	Mobile Phase		Gradient Elution Processes	
		A	B	Time (min)	Mobile B (%)
**Phthalate and nonphthalate plasticizers (positive mode)**				0	40
Luna 3 µm C18 (2) 100Å, 100 × 2.0 mm	0.3	0.1% formic acid in water	0.1% formic acid in methanol	2	40
				4	80
				14	100
				17	100
				20	40
				24	40
**Nonphthalate plasticizers (negative mode)**				0	10
Luna 3 µm C18 (2) 100Å, 100 × 2.0 mm	0.2	4 mM ammonium acetate in water	methanol	2	10
				11.5	100
				22	100
				22.1	70
				25	30
				25.1	10
				28	10

Table S2. Summary of the multiple reaction monitoring (MRM) ion pairs, limit of quantification (LOQ), recoveries from matrix spiking tests, and matrix effects of individual analytes.

Abbreviation	Full name	CAS No	MRM Ions		Recoveries of spiked analytes	Matrix effects	LOQ (ng/g)
			Quatifiers	Qualifiers			
Nonphthalate plasticizers							
ATBC	Acetyl tri-n-butyl citrate	77-90-7	403.2/128.9	403.2/185.2	89 ± 7%	94 ± 7 %	8
ATEC	Acetyl triethyl citrate	77-89-4	319.3/273.1	319.3/157.2	105 ± 8%	99 ± 7%	2
BTHC	n-butyryltri-n-hexyl citrate	82469-79-2	515.4/413.2	515.4/213.0	62 ± 8%	80 ± 9%	1.5
TBC	Tributyl citrate	77-94-1	361.5/185.1	361.5/129.1	99 ± 10%	101 ± 6%	15
TEC	Triethyl citrate	77-93-0	277.3/157.1	277.3/203.1	104 ± 18%	100 ± 7%	2.5
BARO	n-butyl acetyl ricinoleate	140-04-5	397.5/337.1	397.5/263.2	61 ± 4%	79 ± 5%	25
BO	Butyl oleate	142-77-8	339.5/265.2	339.5/247.2	103 ± 16%	99 ± 11%	65
BRO	Butyl ricinoleate	151-13-3	355.5/337.2	355.5/263.2	70 ± 37%	79 ± 16%	20
GMO	Glycerol monooleate	25496-72-4	357.4/265.4	357.4/247.3	127 ± 21%	118 ± 14%	40
MARO	Methyl o-acetylricinoleate	140-03-4	355.4/295.2	355.4/263.2	109 ± 22%	99 ± 15%	35
MO	Methyl oleate	112-62-9	297.4/247.1	297.4/265.3	124 ± 23%	109 ± 7%	65
PO	n-propyl oleate	111-59-1	325.4/265.1	325.4/247.3	102 ± 31%	105 ± 7%	50
THFO	Tetrahydrofurfuryl oleate	5420-17-7	367.4/85.1	367.4/265.3	79 ± 8%	88 ± 9%	10
DEA	Diethyl adipate	141-28-6	203.3/157.1	203.3/111.0	78 ± 9%	89 ± 6%	20
DEHA	Bis(2-ethylhexyl) adipate	103-23-1	371.1/129.1	371.1/101.1	77 ± 9%	80 ± 4 %	8
DHeNoA	Di(n-heptyl,n-nonyl) adipate	68515-75-3	371.0/129.1	371.0/111.1	88 ± 14%	97 ± 9%	70
DiBA	Diisobutyl adipate	141-04-8	259.1/111.0	259.1/129.1	104 ± 5%	127 ± 24%	12
DiDeA	Diisodecyl adipate	27178-16-1	427.1/129.1	427.1/85.1	99 ± 13%	110 ± 10%	12

DMA	Dimethyl adipate	627-93-0	175.3/143.1	175.3/111.1	74 ± 4%	84 ± 7%	75
DnBA	Dibutyl adipate	105-99-7	259.1/111.0	259.1/185.2	82 ± 17%	78 ± 7%	4
DHAZ	Di-n-hexyl azelate	109-31-9	357.5/171.1	357.5/255.2	65 ± 5%	80 ± 6%	5
DiOAZ/DEHAZ	Diisooctyl azelate /Di(2-ethylhexyl) azelate	26544-17-2/103-24-2	413.5/171.1	413.5/283.2	73 ± 13%	87 ± 7%	10
DiDeAZ	Diisodecyl azelate	28472-97-1	469.0/170.9	469.0/71.2	85 ± 18%	120 ± 12 %	12
DMAZ	Dimethyl azelate	1732-10-1	217.1/185.2	217.1/97.1	75 ± 3%	75 ± 14%	35
DBS	Dibutyl sebacate	109-43-3	315.4/241.2	315.4/185.2	94 ± 10%	94 ± 7%	3.5
DEHS	Di(2-ethylhexyl) sebacate	122-62-3	427.5/185.1	427.5/315.3	86 ± 14%	93 ± 5%	5
DMS	Dimethyl sebacate	106-79-6	231.2/199.3	231.2/139.3	109 ± 7%	95 ± 6%	15
DEGDB	Diethylene glycol dibenzoate	120-55-8	315.3/149.1	315.3/105.1	95 ± 2%	96 ± 3%	3.5
DPGDB	Dipropylene glycol dibenzoate	27138-31-4	343.3/163.1	343.3/105.1	77 ± 12%	85 ± 6%	2
TCTM	Tricapryl trimellitate	27251-75-8	547.1/193.0	547.1/323.0	99 ± 14%	111 ± 20%	1.5
THTM	Trihexyl trimellitate	1528-49-0	463.4/277.2	463.4/361.3	92 ± 7%	97 ± 8%	2.5
TiDTM	Triisodecyl trimelliate	36631-30-8	631.1/268.9	631.1/72.8	88 ± 15%	92 ± 18%	14
TiNTM	Triisononyl trimellitate	53894-23-8	589.4/127.2	589.4/319.1	98 ± 22%	99 ± 13%	25
TOTM	Trioctyl trimellitate	3319-31-1	547.2/305.2	547.2/193.1	119 ± 26%	116 ± 8%	2
TMPDDiB	2,2,4-trimethyl-1,3-pentanediol diisobutyrate	6846-50-0	287.4/199.2	287.4/111.2	74 ± 15%	90 ± 4 %	20
TMPDMiB	2,2,4-trimethyl-1,3-pentanediol monoisobutyrate	25265-77-4	217.4/111.1	217.4/69.1	77 ± 13%	86 ± 7%	35
IPMS	Isopropyl myristate	110-27-0	271.4/229.2	271.4/103.1	120 ± 7%	117 ± 7%	25
IPP	Isopropyl palmitate	142-91-6	316.5/257.2	316.5/299.4	60 ± 7%	81 ± 7%	30
DBM/DBF	Di-n-butyl maleate /Dibutyl fumarate	105-76-0/ 105-75-9	229.3/117.1	229.3/57.0	60 ± 8%	74 ± 6%	20
DEHM	Di(2-ethylhexyl) maleate	142-16-5	341.4/117.1	341.4/99.1	77 ± 30%	90 ± 6%	2.5
DESU	Diethyl succinate	123-25-1	175.2/129.1	175.2/101.1	51 ± 15%	74 ± 8%	60
DINCH	Di-isononyl cyclohexane-1,2-dicarboxylate	166412-78-8	425.4/71.1	425.4/155.2	92 ± 15%	127 ± 19%	12
GMS	Glycerol monostearate	31566-31-1	359.4/341.3	359.4/267.0	98 ± 4%	113 ± 18%	2

Phthalate plasticizers							
BBzPh	Butyl benzyl phthalate	85-68-7	313.1/91.1	313.1/149.2	112 ± 10%	130 ± 21%	20
BMPPh	Bis(4-methyl-2-pentyl)phthalate	146-50-9	335.1/149.0	335.1/84.8	97 ± 37%	79 ± 17%	10
DAlPh	Diallyl phthalate	131-17-9	247.1/189.3	247.1/149.0	103 ± 20%	109 ± 33%	12
DAmPh	Diamyl phthalate	131-18-0	307.0/149.1	307.0/65.1	96 ± 23%	120 ± 36%	50
DBPh	Dibutyl phthalate	84-74-2	279.2/149.1	279.2/205.1	126 ± 31%	92 ± 39%	40
DBzPh	Dibenzyl phthalate	523-31-9	347.1/91.0	347.1/181.2	96 ± 13%	118 ± 22%	25
DEPh	Diethyl phthalate	84-66-2	223.0/149.0	223.0/177.0	99 ± 23%	88 ± 30%	8
DEHPh	di(2-ethylhexyl) phthalate	117-81-7	391.4/149.1	391.4/71.2	118 ± 26%	142 ± 26%	8
DHxPh	Dihexyl phthalate	84-75-3	335.1/149.1	335.1/85.1	93 ± 21%	114 ± 16%	4
DiBPh	Diisobutyl phthalate	84-69-5	279.2/149.1	279.2/65.1	87 ± 22%	79 ± 16%	40
DiDPh	Diisodecyl phthalate	26761-40-0	447.1/343	447.1/147.2	91 ± 34%	104 ± 11%	8
DiHePh	Diisoheptyl phthalate	71888-89-6	363.2/149.2	363.2/99.2	96 ± 25%	112 ± 21%	34
DiHxPh	Diisohexyl phthalate	68515-50-4	335.1/148.8	335.1/85.2	76 ± 26%	89 ± 33%	50
DiNPh	Diisononyl phthalate	68515-48-0	419.3/85.1	419.3/220.8	112 ± 39%	126 ± 32%	110
DiPePh	Diisopentyl phthalate	605-50-5	307.2/71.2	307.2/149.1	109 ± 33%	112 ± 24%	10
DiPrPh	Diisopropyl phthalate	605-45-8	251.2/149.1	251.2/65.1	110 ± 19%	90 ± 26%	6
DMPh	Dimethyl phthalate	131-11-3	195.1/163.1	195.1/ 91.1	125 ± 11%	144 ± 33%	80
DMiPh	Dimethyl isophthalate	1459-93-4	195.1/105.0	195.1/91.1	116 ± 19%	113 ± 22%	60
DNPh	Dinonyl phthalate	84-76-4	419.2/149.1	419.2/71.2	122 ± 25%	116 ± 22%	6
DPhPh	Diphenyl phthalate	84-62-8	319.1/225.2	319.1/77.1	88 ± 20%	97 ± 15%	20
DPhiPh	Diphenyl isophthalate	744-45-6	319.0/224.9	319.0/77.1	136 ± 14%	147 ± 34%	8
DPrPh	Di-n-propyl phthalate	131-16-8	251.2/120.9	251.2/42.9	130 ± 15%	143 ± 18%	20
DUPh	Diundecyl phthalate	3648-20-2	475.1/149.0	475.1/120.9	79 ± 15%	88 ± 10%	20
iBCHPh	Isobutylcyclohexyl phthalate	5334-09-8	305.2/149.1	305.2/223.1	71 ± 15%	110 ± 29%	40
Surrogate standards							

DBzPh-d_4	Dibenzylphthalate-d_4	NA	351.0/91.0	351.0/181.0	-
DBPh-d_4	Di-n-butyl phthalate-d_4	93952-11-5	283.1/153.1	283.1/57.1	-
DiBPh-d_4	Di-iso-butyl phthalate-3,4,5,6-d_4	358730-88-8	283.1/153.1	283.1/69.2	-
DCHPh-d_4	Dicyclohexyl phthalate-3,4,5,6-d_4	358731-25-6	335.1/153.1	335.1/171.1	-
DEHP-d_4	Bis(2-ethylhexyl) phthalate-3,4,5,6-d_4	93951-87-2	395.2/153.2	395.2/69.1	-
DEPh-d_4	Diethyl phthalate-3,4,5,6-d_4	93952-12-6	227.1/153.1	227.1/181.1	-
DHxPh-d_4	Di-n-hexyl phthalate-3,4,5,6-d_4	1015854-55-3	339.0/153.2	339.0/89.2	-
DMPh-d_4	Dimethyl phthalate-3,4,5,6-d_4	93951-89-4	199.1/167.1	199.1/81.1	-
DOP-d_4	Di-n-octyl phthalate-3,4,5,6-d_4	93952-13-7	395.2/69.1		-
DPePh-d_4	Di-n-pentyl phthalate-3,4,5,6-d_4	358730-89-9	311.2/153.0	311.2/125.2	-
DPrPh-d_4	Di-n-propyl phthalate-3,4,5,6-d_4	358731-29-0	255.1/153.1	255.1/194.9	-
ATBC-d_3	Acetyl tributyl citrate-d_3	1794753-49-3	406.2/129.0	406.2/139.0	-
DEHA-d_8	Bis(2-ethylhexyl) adipate-d_8	1214718-98-5	379.3/137.3		-
Internal standards					
Coumaphos-d_{10}	Coumaphos-d_{10}	287397-86-8	373.0/228.0		-
tBuP-d_9	tert-butyl paraben-d_9	1216904-65-2	202.0/136.0	202.0/92.0	-

Table S3. Parameters used for the estimation of daily intake via dust ingestion and dermal contact.

Parameters	China		Vietnam		Australia		U.S.		Ref
	Toddlers	Adults	Toddlers	Adults	Toddlers	Adults	Toddlers	Adults	
Indoor exposure fraction (%)	83.3	66.7	83.3	66.7	83.3	66.7	83.3	66.7	2
Body weight (kg)	9.9	62	9.9	62	12.5	75.8	12.3	70	2
Average dust ingestion rate (mg/day)	50	20	50	20	50	20	50	20	2
High dust ingestion rate (mg/day)	200	50	200	50	200	50	200	50	2
Body surface area (cm^2/day)	2564	4615	2564	4615	2564	4615	2564	4615	3
Solid particles adhered onto skin (mg/cm^2)	0.096	0.096	0.096	0.096	0.096	0.096	0.096	0.096	4
Fraction of plastic additives absorbed through the skin[a]	0.000031	0.000031	0.000031	0.000031	0.000031	0.000031	0.000031	0.000031	5
Fraction of plastic additives absorbed through the skin[b]	0.01025	0.01025	0.01025	0.01025	0.01025	0.01025	0.01025	0.01025	5

[a] under low exposure scenario; [b] under high exposure scenario.

Table S4. The reference dose (RfD, µg/kg bw/day) and hazard quotient (HQ) of individual nonphthalate plasticizers with a detection frequency > 90% in at least four regions.

Analytes	NOAEL	LD_{50}	*RfD* calculated[a]	Sources[b]
ATBC	100000		100	ECHA IUCLID
TBC	1000000		1000	ECHA IUCLID
TEC		5900000	590	ECHA IUCLID
GMO		11570000	1157	TEST
MO		16590000	1659	TEST
DEHA	1000000		1000	ECHA IUCLID
DHeNoA		15800000	1580	HPVIS
DiBA		8550000	855	TEST
DnBA		12900000	1290	ECHA IUCLID
DEGDB		2830000	283	ECHA IUCLID
DPGDB		3295000	330	ECHA
TCTM	1000000		1000	TEST
TOTM	1000000		1000	ECHA IUCLID
TMPDDiB	150000		150	ECHA
TMPDMiB	1000000		1000	ECHA IUCLID
IPP		5000000	500	ECHA IUCLID
DBM/DBF		3730000	373	ECHA IUCLID
DEHM		2000000	200	ECHA IUCLID
DINCH			1000[c]	EFSA
GMS	1000000		1000	ECHA IUCLID

[a] RfD calculated = NOAEL (or LD50)/UF, where UF is an uncertainty factor (1000 for NOAEL or 10000 forLD50);[b] NOAEL or RfD values were obtained from the United States Environmental Protection Agency (EPA) Chemistry Dashboard. [c] RfD from literature. For chemicals without proper experimental toxicity data, their toxicities were estimated using the Environmental Protection Agency (EPA) Toxicity Estimation Software Tool (TEST, version 4.2.1).

Table S5. Concentrations (μg/g) of selected nonphthalate plasticizers and phthalate esters in house dust from multiple locations in the Asia-Pacific region and the United States.

	Tianjin, North China			Guangzhou, South China			Hanoi, Vietnam			Adelaide, Australia			Carbondale, U.S.		
	DF	Median	Range	DF	Median	Range	DF	Median	Range	DF	Median	Range	DF	Median	Range
Selected nonphthalate plasticizers[a]															
ATEC	33	nd	nd-0.20	5	nd	nd-0.06	0	nd	nd	44	nd	nd-0.2	47	nd	nd-0.09
BARO	0	nd	nd	0	nd	nd	0	nd	nd	0	nd	nd	12	nd	nd-0.13
MARO	0	nd	nd	nd	nd	nd	0	nd	nd	0	nd	nd	0	nd	nd
THFO	0	nd	nd	nd	nd	nd	0	nd	nd	0	nd	nd	0	nd	nd
DEA	0	nd	nd-0.02	9	nd	nd-0.07	0	nd	nd	2	nd	nd-0.02	12	nd	nd-0.24
DHAZ	0	nd	nd	9	nd	nd-0.12	24	nd	nd-0.03	0	nd	nd	0	nd	nd
DiDeAZ	0	nd	nd	0	nd	nd	0	nd	nd	0	nd	nd	0	nd	nd
DBS	39	nd	nd-0.17	47	nd	nd-0.29	14	nd	nd-0.07	12	nd	nd-0.04	24	nd	nd-0.02
TiDTM	0	nd	nd	0	nd	nd	0	nd	nd	0	nd	nd	0	nd	nd
DESU	33	nd	nd-1.8	5	nd	nd-0.15	0	nd	nd	10	nd	nd-0.28	35	nd	nd-11.2
Phthalate esters															
BBzPh	100	0.14	0.01-3.16	100	0.13	0.03-1.28	100	0.08	0.02-1.15	100	7.33	0.56-156	100	67.6	4.40-161
BMPPh	97	0.08	nd-1.22	95	0.12	nd-2.26	100	0.14	0.05-1.94	100	0.21	0.03-2.79	100	0.59	0.06-43.9
DAlPh	57	0.01	nd-0.23	88	0.04	nd-1.12	38	nd	nd-0.06	93	0.04	nd-0.30	100	0.04	nd-1.0
DAmPh	36	nd	nd-0.14	37	nd	nd-0.84	10	nd	nd-0.29	34	nd	nd-8.77	18	nd	nd-0.2
DBPh	100	21.1	2.46-78.3	100	21.2	8.67-96.5	100	6.19	2.64-48.5	100	8.91	1.24-466	100	10.4	5.79-81.1
DBzPh	33	nd	nd-0.18	60	0.03	nd-0.14	10	nd	nd-0.08	95	0.14	nd-6.11	100	0.95	0.09-29.5
DEPh	100	11.5	2.97-57.3	100	5.0	0.58-29.2	100	5.25	0.47-16.5	100	5.35	1.13-37.8	100	9.84	4.39-40.9
DEHPh	100	208	67.6-651	100	255	83.9-2110	100	119	59.3-645	100	185	26.3-6240	100	219	143-1100
DHxPh	42	nd	nd-0.67	49	nd	nd-2.11	95	0.12	nd-1.73	95	0.19	nd-2.47	100	0.61	0.11-38.6
DiBPh	100	8.69	1.8-35.2	100	42.4	13.8-252	100	3.84	0.98-41.2	100	11.5	1.52-125	100	10.1	5.51-37.2
DiDPh	100	1.38	0.24-8.71	100	0.26	0.04-8.90	62	0.59	nd-4.16	90	2.16	nd-10.4	88	1.52	nd-7.40
DiHxPh	0	nd	nd	0	nd	nd	0	nd	nd	0	nd	nd	0	nd	nd
DiHePh	81	0.33	nd-34.5	98	0.25	nd-1.12	95	0.06	nd-0.42	98	0.74	nd-19.3	100	2.17	0.55-31.0
DNPh	25	nd	nd-3.37	5	nd	nd-0.32	0	nd	nd	12	nd	nd-0.57	0	nd	nd
DiNPh	100	71.7	4.37-536	100	104	13.6-879	100	8.7	2.22-71.5	100	40.8	5.01-470	100	96.8	22.4-167
DiPePh	58	0.02	nd-0.21	98	0.07	nd-0.49	33	nd	nd-0.06	76	0.05	nd-1.57	71	0.05	nd-0.20
DiPrPh	100	0.07	0.01-0.44	95	0.03	nd-0.44	95	0.02	nd-0.14	100	0.09	0.01-0.72	100	0.35	0.05-5.69
DMPh	94	0.31	nd-10.8	98	0.74	nd-7.91	62	0.09	nd-1.88	90	0.4	nd-3.70	100	1.17	0.08-5.76
DMiPh	22	nd	nd-0.28	0	nd	nd	0	nd	nd	0	nd	nd	0	nd	nd
DPhPh	0	nd	nd	0	nd	nd	0	nd	nd	0	nd	nd	0	nd	nd
DPhiPh	0	nd	nd	0	nd	nd	0	nd	nd	0	nd	nd	0	nd	nd
DPrPh	0	nd	nd	0	nd	nd	0	nd	nd	0	nd	nd	0	nd	nd
DUPh	92	0.57	nd-15.9	100	1.26	0.54-8.71	90	0.08	nd-1.04	100	0.48	0.04-6.82	100	1.43	0.21-2.67

iBCHPh	11	nd	nd-0.16	28	nd	nd-0.54	19	nd	nd-0.09	61	0.05	nd-0.52	53	0.04	nd-0.11
Σ24PAEs		496	132-1880		520	211-1720		153	73.0-825		295	41.8-7380		480	255-1410

[a] chemicals with a detection frequency < 50% in all countries.

Table S6. Estimated daily intakes (EDIs, ng/kg bw/day) of nonphthalate plasticizers via indoor dust for toddlers and adults in the Asia-Pacific region and the United States.

Regions			Dust ingestion	Dermal contact	Sum
Tianjin, North China	Toddlers	Average/low	837 (42.1-5510) [a]	0.13 (0.006-0.84) [b]	837 (42.1-5510)
		High	3350 (168-22050)	42.3 (2.12-278)	3390 (170-22330)
	Adults	Average/low	42.8 (2.15-282)	0.03 (0.001-0.19)	42.8 (2.15-282)
		High	107 (5.38-705)	9.72 (0.49-64.0)	117 (5.87-769)
Guangzhou, South China	Toddlers	Average/low	1060 (395-3880)	0.16 (0.06-0.59)	1060 (395-3880)
		High	4240 (1580-15520)	53.5 (20.0-196)	4290 (1600-15720)
	Adults	Average/low	54.2 (20.2-198)	0.04 (0.01-0.14)	54.2 (20.2-198)
		High	136 (50.6-496)	12.3 (4.59-45.0)	148 (55.2-541)
Hanoi, Vietnam	Toddlers	Average/low	74.9 (6.52-854)	0.01 (0.001-0.13)	74.9 (6.52-854)
		High	300 (26.1-3420)	3.78 (0.33-43.1)	304 (26.4-3460)
	Adults	Average/low	3.83 (0.33-43.7)	0.003 (0.0002-0.03)	3.83 (0.33-43.7)
		High	9.57 (0.83-109)	0.87 (0.08-9.92)	10.4 (0.91-119)
Adelaide, Australia	Toddlers	Average/low	383 (90.0-2490)	0.06 (0.01-0.38)	383 (90.0-2490)
		High	1530 (360-9970)	19.3 (4.54-126)	1550 (365-10100)
	Adults	Average/low	20.2 (4.75-132)	0.01 (0.003-0.09)	20.2 (4.75-132)
		High	50.6 (11.9-329)	4.60 (1.08-29.9)	55.2 (13.0-359)
Carbondale, U.S.	Toddlers	Average/low	393 (86.7-752)	0.06 (0.01-0.11)	393 (86.7-752)
		High	1570 (347-3010)	19.8 (4.37-37.9)	1590 (351-3050)
	Adults	Average/low	22.1 (4.88-42.3)	0.02 (0.003-0.03)	22.1 (4.88-42.3)
		High	55.3 (12.2-106)	5.02 (1.11-9.61)	60.3 (13.3-116)

[a] represents the average estimated level of dust ingestion. [b] represents the low estimated level through dermal contact.

Table S7. The median hazard quotient (HQ) of nonphthalate plasticizers in the Asia-Pacific region and the United States.[a]

	Tianjin, North China		Guangzhou, South China		Hanoi, Vietnam		Adelaide, Australia		Carbondale, U.S.	
	Toddler	Adult	Toddler	Adult	Toddler	Adult	Toddler	Adult	Toddler	Adult
ATBC	5.32E-04	1.83E-05	1.23E-03	4.23E-05	2.73E-05	9.39E-07	3.28E-04	1.17E-05	1.60E-04	6.08E-06
TBC	8.52E-06	2.93E-07	9.20E-06	3.17E-07	3.41E-07	1.17E-08	4.72E-06	1.68E-07	4.94E-06	1.87E-07
TEC	1.44E-06	4.97E-08	2.89E-06	9.95E-08	NA	NA	2.29E-06	8.13E-08	7.21E-06	2.73E-07
GMO	7.23E-05	2.49E-06	8.51E-04	2.93E-05	1.41E-04	4.86E-06	7.86E-04	2.80E-05	2.47E-04	9.34E-06
MO	4.68E-05	1.61E-06	1.50E-04	5.16E-06	1.16E-05	4.00E-07	2.13E-04	7.58E-06	2.30E-04	8.71E-06
DEHA	2.73E-06	9.39E-08	2.40E-05	8.27E-07	8.52E-07	2.93E-08	6.75E-06	2.40E-07	2.33E-06	8.83E-08
DHeNoA	2.05E-06	7.06E-08	3.04E-05	1.05E-06	3.34E-06	1.15E-07	8.46E-06	3.01E-07	3.56E-06	1.35E-07
DiBA	2.23E-05	7.69E-07	7.41E-05	2.55E-06	2.43E-05	8.37E-07	1.20E-04	4.25E-06	5.09E-05	1.93E-06
DnBA	1.24E-05	4.28E-07	8.98E-06	3.09E-07	3.96E-07	1.36E-08	4.18E-07	1.49E-08	2.13E-07	8.06E-09
DEGDB	1.69E-05	5.81E-07	2.47E-05	8.50E-07	NA	NA	1.48E-05	5.26E-07	3.54E-05	1.34E-06
DPGDB	7.64E-05	2.63E-06	7.80E-05	2.68E-06	NA	NA	1.05E-04	3.74E-06	9.19E-05	3.48E-06
TCTM	1.74E-04	5.98E-06	8.64E-04	2.97E-05	1.87E-06	6.45E-08	2.70E-06	9.60E-08	8.92E-06	3.38E-07
TOTM	9.87E-05	3.40E-06	5.06E-04	1.74E-05	2.90E-06	9.97E-08	1.62E-06	5.76E-08	3.70E-06	1.40E-07
TMPDDiB	5.23E-05	1.80E-06	1.48E-05	5.09E-07	NA	NA	1.26E-05	4.48E-07	2.10E-05	7.97E-07
TMPDMiB	2.20E-05	7.57E-07	6.65E-06	2.29E-07	6.82E-07	2.35E-08	2.92E-05	1.04E-06	5.40E-05	2.05E-06
IPP	1.64E-05	5.63E-07	2.15E-05	7.39E-07	NA	NA	6.75E-06	2.40E-07	9.05E-06	3.43E-07
DBM/DBF	2.51E-05	8.65E-07	3.24E-05	1.12E-06	NA	NA	5.68E-05	2.02E-06	6.91E-05	2.62E-06
DEHM	7.67E-06	2.64E-07	3.41E-06	1.17E-07	2.56E-07	8.80E-09	2.02E-06	7.20E-08	4.80E-06	1.82E-07
DINCH	4.26E-05	1.47E-06	1.96E-04	6.75E-06	1.31E-05	4.52E-07	1.36E-05	4.85E-07	4.11E-06	1.56E-07
GMS	1.61E-04	5.54E-06	1.04E-04	3.59E-06	2.15E-05	7.39E-07	8.12E-05	2.89E-06	6.57E-05	2.49E-06
HI	1.39E-03	4.80E-05	4.23E-03	1.46E-04	2.50E-04	8.60E-06	1.80E-03	6.39E-05	1.07E-03	4.07E-05

[a] Only chemicals with a detection frequency > 90% in at least four regions are calculated; NA: not available; HI: hazard index, it was calculated by summing the HQs for individual analytes.

References

1. Xie, Q.; Guan, Q.; Li, L.; Pan, X.; Ho, C. L.; Liu, X.; Hou, S.; Chen, D. Exposure of children and mothers to organophosphate esters: Prediction by house dust and silicone wristbands. *Environ. Pollut.* **2021**, *282*, 117011.
2. Tan, H.; Yang, L.; Huang, Y.; Tao, L.; Chen, D. "Novel" Synthetic Antioxidants in House Dust from Multiple Locations in the Asia-Pacific Region and the United States. *Environ. Sci. Technol.* **2021**, *55*, 8675–8682.
3. Huang, Y.; Tan, H.; Li, L.; Yang, Y.; Sun, F.; Li, J.; Gong, X.; Chen, D. A broad range of organophosphate tri- and di-esters in house dust from Adelaide, South Australia: Concentrations, compositions, and human exposure risks. *Environ. Int.* **2020**, *142*, 105872.
4. Johnson-Restrepo, B.; Kannan, K. An assessment of sources and pathways of human exposure to polybrominated diphenyl ethers in the United States. *Chemosphere*, **2009**, *76*, 542−548.
5. Giovanoulis, G.; Bui, T.; Xu, F.; Papadopoulou, E.; Padilla-Sanchez, J. A.; Covaci, A.; Haug, L. S.; Cousins, A. P.; Magner, J.; Cousins, I. T.; de Wit, C. A. Multi-pathway human exposure assessment of phthalate esters and DINCH. *Environ. Int.* **2018**, *112*, 115−126.
